# Time-Domain NMR: Generating Unique Insights into the Characterization of Heterogeneous Catalysis in Liquid Phase

**DOI:** 10.1021/acscatal.4c04789

**Published:** 2025-01-21

**Authors:** Murilo T. Suekuni, Carmine D’Agostino, Alan M. Allgeier

**Affiliations:** † Department of Chemical and Petroleum Engineering, Center for Environmentally Beneficial Catalysis, and Wonderful Institute for Sustainable Engineering, University of Kansas, Lawrence, Kansas 66045, United States; ‡ Department of Chemical Engineering, University of Manchester, Manchester M13 9PL, U.K.; § Dipartimento di Ingegneria Civile, Chimica, Ambientale e dei Materiali (DICAM), Alma Mater Studiorum − Università di Bologna, Via Terracini 28, 40131 Bologna, Italy

**Keywords:** time-domain NMR, adsorption, diffusion, relaxometry, in situ

## Abstract

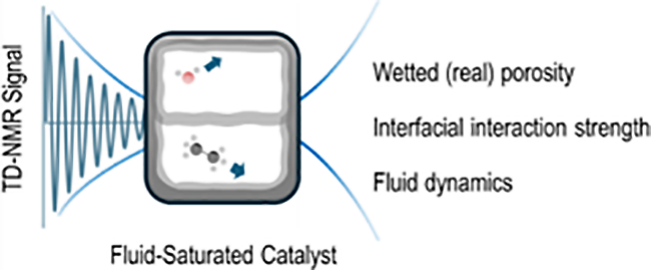

Time-domain (TD) nuclear magnetic resonance (NMR) comprises a family of tools for characterizing wetted porosity and surface area, fluid-catalyst surface adsorption energy, liquid distribution in packed beds, and transport of fluids in catalyst materials. These methods are differentiated from NMR spectroscopy in that the data are not analyzed in the frequency domain and often benefit from the use of low magnetic field strength. The increased accessibility of commercial, low-field, benchtop NMR instruments has supported substantial growth in TD NMR research in catalysis. This perspective offers a tutorial on physical phenomena critical to TD NMR methods, a summary of applications in both ex situ and in situ settings, and commentaries on ensuring experimental rigor and opportunities for growth in the field. The unique insights accessible from TD NMR often cover length scales in the tens of nanometers to tens of micrometers and are complementary to other catalyst characterization methods probing molecular structure and identity.

## 1 Introduction

Nuclear magnetic resonance (NMR) phenomena were initially described in the 1930s, with Isidor Rabi receiving the 1944 Nobel Prize in Physics^1^ for discovering that certain atomic nuclei absorb specified (quantized) energies in the radiofrequency spectrum when subject to an external magnetic field. Felix Bloch and Edward Mills Purcell harnessed the physical phenomenon and applied precise measurements of magnetic field strength and radio wave energy to extract the composition of liquid and solid analytes, garnering the 1952 Nobel Prize in Physics.^2^ This resonance phenomenon has grown beyond an intriguing portion of experimental physics to the basis of characterization tools impacting diverse fields across the full spectrum of science, engineering, and medicine.^3^ Authoritative reviews of applications to catalysis,^4^ acid site characterization^5^ and operando characterization of catalytic reactions,^6^ have recently appeared but primarily cover applications of NMR spectroscopy. Indeed, for many investigators of catalytic phenomena, NMR is synonymous with spectroscopy, which provides invaluable insights into the chemical structure and kinetics of reactions by integration of specific spectral peaks as a function of the extent of reaction. Fundamentally, NMR signals decay with time, and measurements in the time-domain (TD) predate spectroscopy.^2^ The recent expansion of high-quality, low-field, permanent magnet-based NMR instrumentation has renewed the catalysis community’s interest in the application of time-domain methods in the field of catalysis. Furthermore, advances in instrumentation have enabled full-packed bed reactors to be characterized by operando TD NMR techniques.

TD NMR methods comprise the measurement of spin–lattice (*T*_1_) and spin–spin (*T*_2_) relaxation time constants, in addition to applications of pulsed-field gradients for characterizing self-diffusivity (*D*), vide infra. While NMR spectroscopy predominantly relies upon the Fourier transform of time-domain data to generate molecular structure of analytes, TD NMR provides insight into phenomena such as molecular translation and rotation.^7^ Catalysis is dependent upon molecular transport to active sites, and numerous investigations benefit from characterization by TD NMR methods, Table 1. Since many heterogeneous catalysts comprise nanoporous solids, quantitation of restricted transport in the liquid phase and the role of surface composition upon that transport are critical to understanding reaction performance. The impact of fluid confinement on NMR relaxation rates also presents a convenient basis for characterizing porous media, which host such fluids. The quantitative nature of the NMR response further strengthens its utility in several catalysis studies. This Perspective is organized in sections, which (1) offer a review of NMR physics, (2) provide a description of quantitative ex situ methods, which deliver fundamental insights into catalyst performance, (3) summarize emerging operando TD NMR methods yielding information not attainable by traditional catalysis characterization tools, (4) offer our advice on rigorous experimentation and data analyses, and (5) give a perspective on areas of growth for this field.

**Table 1:**
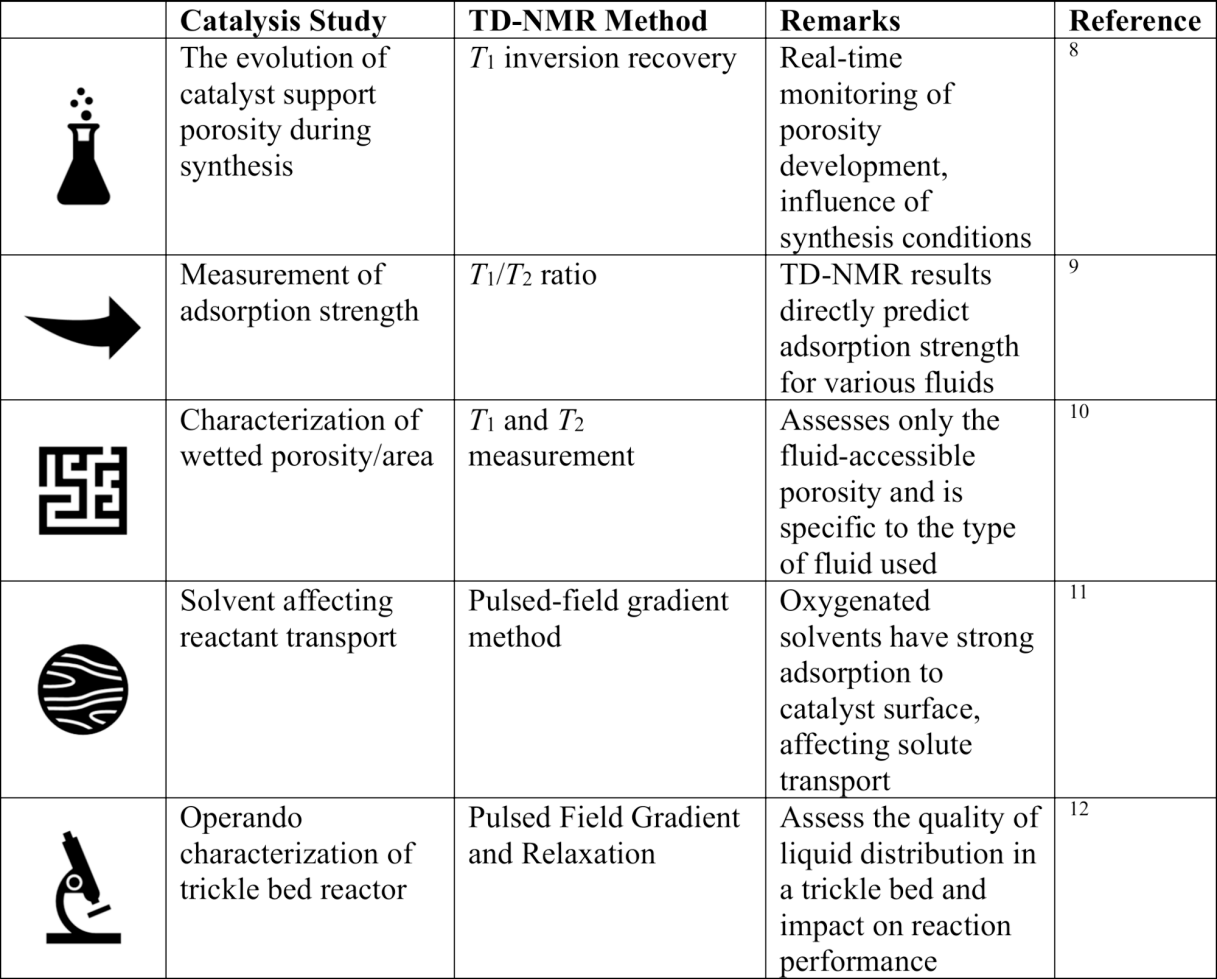
What Can We Study with TD NMR in Catalysis?−

### 1.1 Nuclear Magnetic Resonance Fundamentals

**Figure 1:**
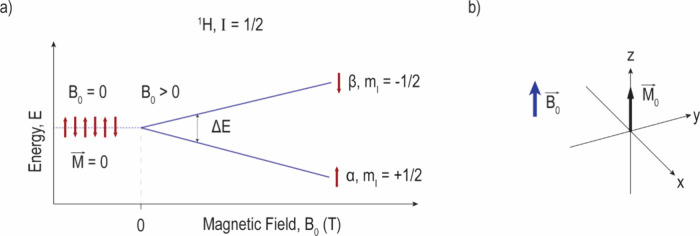
(a) Nuclear Zeeman splitting of ^1^H under the influence of an externally applied magnetic field (**B**_0_), where a twofold degeneracy comprising spins at high- and low-energy states is observed, and (b) representation of the resulting magnetization vector along the positive *z* direction due to the slight excess of low-energy spins.

Nuclear magnetic resonance exploits the nature of certain nuclei with a nonzero spin number (*I*).^13,14^ In the quantum domain, an atomic nucleus is characterized by the orientation and magnitude of its intrinsic properties, such as angular momentum (**L**) and magnetic moment (**μ**).^14^ At an undisturbed state, a given spin population possesses a total bulk magnetization (**M** = ∑**μ**) of approximately zero, resulting from the random **μ** distribution.^15^ During an NMR experiment, an externally applied magnetic field (**B**_0_) in the positive *z* direction introduces energy (*E*_*z*_) to the spin system and is mathematically expressed by the scalar product of **μ** and **B**_0_ (eq 1):
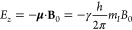
1where *h* represents Planck’s constant (6.6261 × 10^–34^ J s), and *m*_*I*_ and γ are the nucleus azimuthal quantum number and gyromagnetic ratio, respectively.^14^ The applied energy induces a (2*I* + 1)-fold energetic division in a phenomenon known as Zeeman splitting (Figure 1). Commonly studied NMR-active isotopes, such as ^1^H and ^13^C, possess *I* = 1/2, resulting in a two-state split.^16^ These conditions are characterized by low-energy (α, *m*_*I*_ = +1/2) and high-energy (β, *m*_*I*_ = −1/2) states (or Zeeman levels).^13,14^ The electromagnetic-induced transition between energy states is referred to as *nuclear magnetic resonance*, and their energy difference (Δ*E*_*z*_) is calculated via eq 2:^13,14^
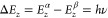
2where ν represents the frequency of the applied electromagnetic radiation. At thermal equilibrium, the ratio of the spin populations at α (*N*_α_) and β (*N*_β_) states is represented by the Boltzmann distribution (eq 3):^14^
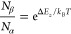
3where *k*_B_ is the Boltzmann constant (1.3806 × 10^–23^ m^2^ kg s^–2^ K^–1^) and *T* is the temperature. In the α (or spin-up) state, **μ** is in parallel alignment with **B**_0_, while for the β (or spin-down) state, **μ** and **B**_0_ are antiparallel to each other.^13^ Under common experimental conditions, there is a small excess of low-energy state spins, resulting in an overall polarization along **B**_0_, Figure 1b. The resolution of NMR experiments is influenced by the gyromagnetic ratio of the nucleus, its concentration in the sample, and the magnetic field strength.^14^ The proton, ^1^H, is commonly chosen due to its natural abundance, large γ value, and presence in the structure of many substances of interest.^14^

#### 1.1.1 Radiofrequency Energy and Relaxation

**Figure 2:**
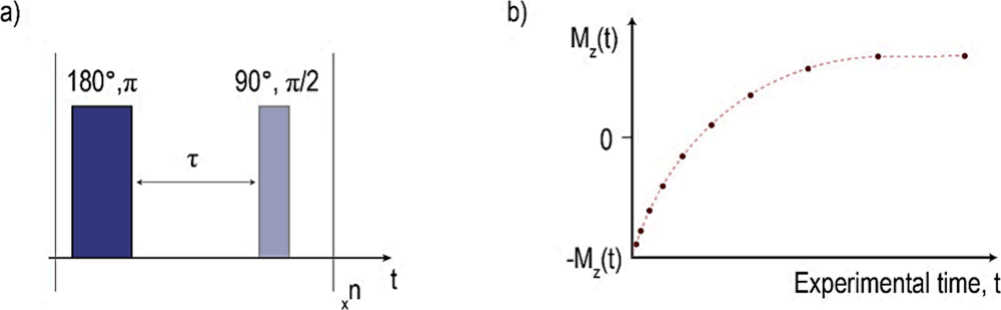
(a) Representation of the inversion recovery pulse sequence, where combinations of 180° and 90° radiofrequency pulses are applied *n* times during the experiment, and (b) the resulting longitudinal magnetization recovery plot over the experimental time, *t*.

**Figure 3:**
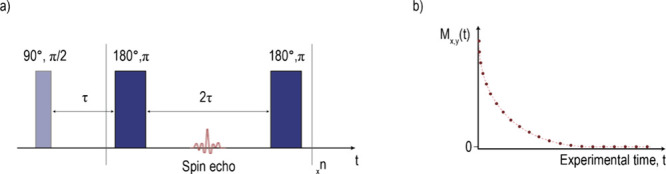
(a) Representation of the CPMG pulse sequence, where equally spaced 180° radiofrequency pulses are applied *n* times after an initial 90° pulse, and (b) graphical representation of the resulting magnetization decay in the transverse plane.

At equilibrium, the spin populations in a homogeneous magnetic field will show a precessional motion about **B**_0_ at a nucleus-specific angular frequency (Larmor frequency, ω_0_) (eq 4):^17^
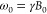
4In NMR experiments, radiofrequency pulses are applied perpendicular to **B**_0_ to manipulate the orientation of the spins, consequently impacting the total sample magnetization (**M**_0_). A π/2 radian (or 90°) pulse induces a 90° rotation of **M**_0_, transferring the total magnetization to the transverse (*xy*) plane.^14^ At the same conditions, if the pulse duration is doubled, a π radian (or 180°) pulse, results in the magnetization transfer onto the negative *z* axis.^14^ As **B**_0_ is held constant along the positive longitudinal axis, the system will return to its initial thermal equilibrium state by redistributing the energy excess in a phenomenon called *relaxation*.^14,18^ Nuclei relaxation rates may reflect several system properties, including molecular motion dynamics, temperature variation, and the presence of magnetization sinks.^13,14^ The spin–lattice or longitudinal relaxation time (*T*_1_) characterizes the restoration of magnetization in the *z* axis and the rate of magnetic energy transfer from the nuclei to the system (lattice). Generally, *T*_1_ is obtained using the inversion recovery pulse sequence, introduced by Erwin Hahn in 1949,^19^Figure 2. In this method, a π radian pulse transfers the total magnetization into the negative *z* axis and is followed by a π/2 radian pulse after a certain spacing period (τ).^20^ The latter transfers the remaining magnetization into the transverse plane, and a signal is detected.^20^ Ultimately, *T*_1_ is measured by repeating this sequence with increasing τ values, spaced out by a recovery delay time. The longitudinal magnetization build-up follows an approximate exponential behavior (eq 5):^13,14^
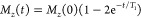
5where *M*_*z*_(*t*) is the longitudinal magnetization intensity at a given experimental time (*t*), and *M*_*z*_(0) is the initial longitudinal magnetization. Simultaneous with the magnetization recovery in the *z*-direction, the spin system loses coherence in the *x*–*y* plane, characterized by the transverse or spin–spin relaxation time, *T*_2_.^18^ Conventionally, *T*_2_ values are measured using the Carr–Purcell–Meiboom–Gill (CPMG) pulse sequence, elaborating the work by Erwin Hahn, on the detection of spin echoes (Hahn Echo) from the application of 90° and 180° pulses.^21−23^ The CPMG sequence consists of the application of a π/2 radian pulse followed by consecutive, equally spaced π radian pulses (Figure 3a). Similar to the longitudinal magnetization growth, the signal decay in the transverse plane follows an exponential behavior (Figure 3b). This process is represented by a sinusoidal plot of the magnetization intensity over experimental time, known as the free induction decay (FID). The FID proceeds with a time constant, *T*_2_^*^, which is shorter than *T*_2_ and comprises contributions from the disruption in phase coherence of NMR spins and inhomogeneities in the magnetic field.^24^ The impact of the latter (magnetic field inhomogeneities) on *T*_2_ is addressed by the 180° pulses that refocus groups of fast and slow precessing spins.^20^ The value of *T*_2_ is determined at the period when the transverse magnetization intensity (*M*_*xy*_(*t*)) equals 36.8% of the initial magnetization and is calculated via eq 6:^25^
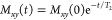
6where *M*_*xy*_(0) is the initial magnetization in the *xy* plane, i.e., immediately after the π/2 radian pulse. The indirect exchange of energy during the spin–lattice and spin–spin relaxation leads to a complex relationship between *T*_1_ and *T*_2_, where *T*_1_ is always ≥ *T*_2_.^14^ Compared to spectroscopy, which requires the Fourier transformation of the acquired signal for chemical speciation, this Perspective comprises data collected and analyzed in the time domain.

In addition to NMR relaxation times, pulsed-field gradient (PFG) experiments are assets for studying mass transport phenomena in heterogeneous catalysts and other porous media.^26−31^ This technique provides the self-diffusion coefficient of fluid molecules, which are perturbed by pore entrapment and surface adsorption.^31^ Experimentally, a gradient pulse with a certain magnetic strength (*g*) applied in the *z* direction generates an additional inhomogeneous field that superimposes **B**_0_ over a certain period (δ).^27,29^ The applied gradient field makes the spin orientation spatially dependent. After a determined time interval (Δ), a second identical gradient pulse offsets the effects of the first and restores homogeneity for spins that remained unmoved.^27,29^ Conversely, molecules that relocated over a certain distance in the longitudinal direction (*d*_*z*_) across the field gradient will experience a phase shift equal to γ*gd*_*z*_δ.^27,29^ The diffusion-attenuated NMR signal, ψ, is associated to molecular diffusion via eq 7:^28,29^
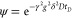
7where *t*_D_ is the diffusion observation time in PFG experiments and γ is the gyromagnetic ratio, as previously defined. The *D* values are determined by measurements of ψ in response to *g*, δ, and *t*_D_(28) and are particularly relevant in characterizing restricted diffusion of fluids confined in the pores of catalysts.

### 1.2 Relaxation Mechanisms and Porous Media Characterization via Time-Domain NMR

**Figure 4:**
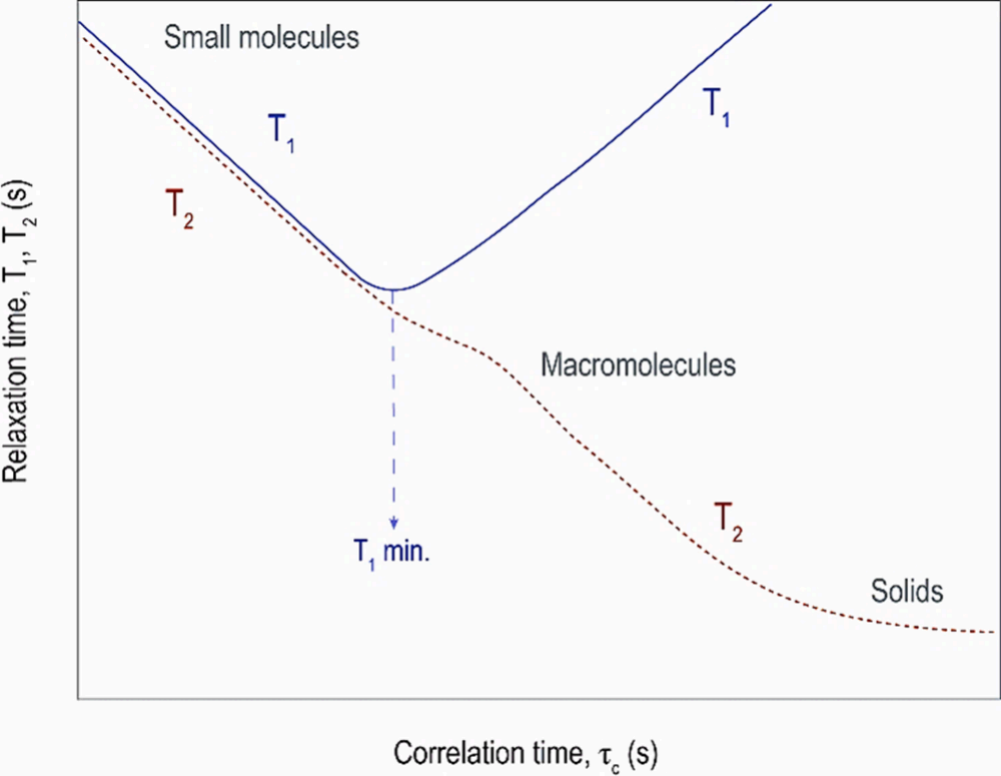
Relationship between relaxation and correlation times. The solid blue line represents *T*_1_ and the dark red line *T*_2_. In systems characterized by short correlation times, such as small molecules or nonviscous fluids, *T*_1_ and *T*_2_ are nearly identical. As molecular size (and correlation time) increases, *T*_1_ reaches its minimum and the two curves separate, where *T*_1_ continues to increase and *T*_2_ decreases. Reproduced with permission from ref (33). Copyright 1948 American Physical Society.

The observed relaxation times offer valuable information about surface-fluid interaction and mass transport in porous systems. Magnetic fluctuations in a sample reflect the many interactions occurring in the spin system. These energetic exchanges are associated with the relaxation rates of molecules via correlation times (τ_c_), which represent the lifetime of a nucleus in a given state.^32^ Pfeifer comprehensively reviewed the relationship between relaxation mechanisms and molecular interactions, shortly described as follows.^32^ In simple, pure systems, relaxation may occur via intramolecular interactions and molecular tumbling, e.g., rotational and translational motion. In this case, the relaxation rates are associated with τ_c_ via eqs 8 and 9:^33^
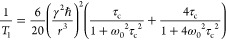
8
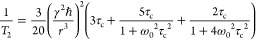
9where ℏ is the reduced Planck’s constant (*h*/2π) and *r* is the distance between two neighboring nuclei. Bloembergen, Purcell, and Pound further explored the relationship between NMR relaxation and molecular mobility^33^ (Figure 4). Assuming a spherical shape, the authors correlated the molecule radius (*a*) and the system viscosity (η) with τ_c_ through the Debye model (eq 10):^33^
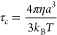
10

For highly mobile small molecules, ω_0_τ_c_ ≪1, and *T*_1_ ≈ *T*_2_. As mobility declines and τ_c_ increases, *T*_1_ decreases to a minimum at ω_0_τ_c_ ≈ 1.0. As τ_c_ continues to increase until ω_0_τ_c_ ≫1.0, *T*_1_ starts to increase again while *T*_2_ decreases continuously.^33,34^ Solids with their very large τ_c_ are often undetected in transverse relaxation analysis of multiphase samples, such as particle suspensions.^10,35−42^ Surface-adsorbed fluid molecules will have restrained mobility and will experience faster relaxation than those in the bulk phase.^15^ In this case, solvent relaxation may occur via intermolecular interactions, such as hydrogen bonding, homonuclear dipolar coupling, or electron–proton interactions (paramagnetic influence).^32^ Hydrogen-bonded molecules have a long residence time on surface hydroxyl groups (τ_OH_), allowing their modeling via eqs 8 and 9, assuming τ_c_ ≈ τ_OH_.^32^ Conversely, relaxation via homonuclear (^1^H–^1^H) dipolar couplings was described by Henry Torrey (eqs 11 and 12):^32,43^
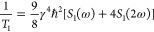
11
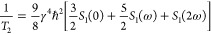
12Assuming that the mean molecular flight, i.e., translational diffusion jump, is much longer than the distance between two protons, *S*_1_(ω) is described by eq 13:
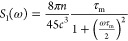
13where *n* represents the proton density (bulk and surface-adsorbed), τ_m_ is the mean time between molecular flights, and *c* is the closest approach distance between two nuclei.^32,43^ Relaxation via electron–proton interactions may dominate the signal depending on the concentration of paramagnetic substances.^44,45^

#### 1.2.1 Relaxation via Electron–Proton Interactions

Paramagnetism emerges from unpaired electrons in organic molecules, e.g., free radicals, or in molecular orbitals of certain elements, e.g., transition metals. Electron–proton interactions induce relaxation and are independent of temperature.^45^ The fluctuating magnetic field generated by the unpaired electrons and their fast relaxation (10^5^ to 10^13^ times faster than the proton ^1^H) significantly shortens the observed *T*_1_ and *T*_2_ of interacting fluids, which is critical in geological exploration and medical applications of magnetic resonance imaging.^45−50^ For catalysis, the relaxation enhancement caused by dissolved paramagnetic metal species depends on the coordination of proton-bearing molecules with the metallic center, where inner-sphere interactions are significantly stronger than outer-sphere.^48^ Insoluble paramagnetic metal particles also promote fast solvent relaxation in solid suspensions and fluid-saturated media, depending on their concentration.^37,47,51,52^ For example, trace amounts of Fe, Mn, Cu, and Ni, which are commonly used in heterogeneous catalysis, can have a significant influence on NMR measurements.^45,48^ Several works have shown how surface-adsorbed metals enhance solvent relaxation. Bryar and co-workers studied how the concentration of Fe species altered the surface relaxivity of fluid-saturated quartz sand and silica, comparing systems with adsorbed and free Fe at varying pH.^51^ Bryar and Knight discussed the impacts of Fe oxidation states on the surface relaxivity of aqueous soil suspensions.^44^ Dissolved oxygen had negligible impacts on the relaxation of bulk water; however, as Fe^2+^ was oxidized to Fe^3+^, the authors reported a significant reduction in the measured *T*_1_. Zhu and co-workers elucidated how the longitudinal surface relaxivity of zirconia and silica nanoparticles increased upon the adsorption of Fe^3+^ ions.^52^ Recently, Allgeier and co-workers elucidated the impacts of iron impurities when using TD NMR to determine the wetted surface area in polymer particle suspensions.^37^ Without correcting for the influence of Fe, the authors showed that the wetted surface areas could be overestimated by factors higher than 3. In heterogeneous catalysis applications, D’Agostino et al. investigated the influence of paramagnetic impurities in the relaxation times of 1-octanol in Al_2_O_3_ samples doped with CuSO_4_.^53^ The authors reported that while both longitudinal and transverse relaxation rates increased proportionally to the concentration of paramagnetic species, the *T*_1_/*T*_2_ data remained largely unaffected. These findings indicate that the relaxation time ratios are more strongly influenced by reduced molecular mobility due to confinement inside pores and surface interactions than the influence of paramagnetic relaxation sinks. In follow-on research, two-dimensional *T*_1_–*T*_2_ maps characterized changes in surface adsorption sites of 1-octene in a similar porous matrix, i.e., CuSO_4_-doped Al_2_O_3_.^54^ In this study, the signals in relaxation time maps were attributed to individual intermolecular interactions between 1-octene and alumina or copper sulfate sites, providing insights into fluid-surface affinity and indicating a higher affinity between 1-octene and Al_2_O_3_ than CuSO_4_. Understanding electron–proton relaxation is important for designing and optimizing heterogeneous catalysts containing paramagnetic metals. Further research on the quantitative aspects of TD NMR application to these systems should provide fundamental insights into fluid transport in porous media, the accessibility of active sites to reactants and solvents, and the strength of interfacial interactions.

#### 1.2.2 Correlation of Relaxation Times to Surface Adsorption

Reflecting on eqs 11–13 and recognizing that τ_m_ is reflective of a diffusion process that is temperature-dependent, Torrey^43^ noted the relationship shown in eq 14:
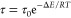
14where τ_0_ is the reference correlation time, Δ*E* is the activation energy for the process, *R* is the gas constant, and *T* is the absolute temperature. For a system of molecules interacting with a surface, wherein nuclear spin relaxation is dominated by molecule–surface interactions of like spins (notably this excludes the influence of unpaired electrons), two correlation times are relevant: that of diffusional hopping, τ_m_, and that for exchange with the bulk, τ_s_, each with a dependence on temperature and activation energy. The τ_s_/τ_m_ ratio has been correlated to surface affinity.^55^ It becomes convenient to ratio *T*_1_/*T*_2_, as the prefactors cancel for low-field NMR (eq 15):
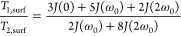
15The spectral density, *J*(ω), takes the form of eq 16, which for a constant temperature and field strength depends only on τ_m_ and τ_s_^9,56−58^ and correlates to the surface adsorption strength:
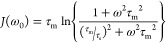
16Indeed, D’Agostino et al. have recommended the dimensionless term *e*_surf_ = −*T*_2_/*T*_1_ as a surrogate for surface adsorption energies providing important insight into catalyst behaviors.^57^

Fast-field-cycling (FFC) NMR provides additional insights into molecular dynamics.^59^Equation 13 relevant to homogeneous (bulk) fluid suggests that in the limit where (*ωτ*_*m*_)^2^ ≪ 1 spectral density is not significantly dependent upon field-strength and the correlated Larmor frequency, ω. When imbibed in a porous medium with opportunity for surface to fluid dipole–dipole interactions, we see from eq 16 that varying field-strength (and ω) can have a notable influence on spectral density of the fluid and thus *T*_1_. In fast-field-cycling relaxometry, *T*_1_ is measured for a range of magnetic field strengths and associated frequencies (kHz to 100 MHz). A plot of the spin–lattice relaxation rate (*R*_1_ = 1/*T*_1_) versus Larmor frequency (labeled the NMR distribution curve, NMRD) is interpreted in light of molecular dynamics. Some authors utilize a monoexponential fit to the decay data,^60^ but data have been fit to multiple populations using regularization methods for additional insights.^61^

#### 1.2.3 Characterization of Porous Media via TD NMR

TD NMR is a convenient characterization method for porous media that avoids aggressive sample preparation and analysis conditions.^37^ For fluid-saturated porous materials, the observed transverse relaxation rates may comprise the signal from the bulk fluid (1/*T*_2b_), surface-adsorbed molecules (1/*T*_2s_), and diffusion-related contributions (1/*T*_2D_) (eq 17):^62,63^
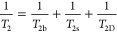
17Some authors adopt a two-site model (surface versus bulk) invoking an alternative to eq 17 that includes population fractions.^14^ For eq 17 the second term acknowledges that fluid-surface dipole–dipole interactions accelerate NMR decay and expresses a combination of the impacts from the surface-to-volume ratio (*S*/*V*) of the pore (and thus the frequency of surface fluid interactions) and its chemistry through the surface relaxivity parameter (ρ_2_) (eq 18):^62,64^
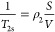
18In the absence of paramagnetic compounds and with knowledge of *S*/*V*, ρ_2_ is directly associated with the affinity of the solid–fluid pair.^15,41,65^ The diffusion component emerges from inhomogeneities in the magnetic field caused by differences in magnetic susceptibility (χ) between the surface and the fluid.^62^ The values characterize the magnetic nature of a nucleus.^13^ Diamagnetic nuclei possess χ > 0 and weakly repel an externally applied magnetic field. Conversely, paramagnetic centers have χ < 0 and pull the field inward, introducing inhomogeneities.^13^*T*_2D_ can be expressed as shown in eq 19:
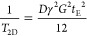
19where *G* represents the strength of the magnetic field gradient, *t*_E_ is the experimental echo spacing, and *D* is the self-diffusion coefficient of liquid molecules, as previously defined. The contributions from diffusion are minimized by using low magnetic field strengths and short *t*_E_.^62^ Hence, eq 17 can be expressed as eq 20:
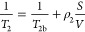
20

The longitudinal relaxation rate (1/*T*_1_) is described by an identical form of eq 20.^62^ The direct correlation between eq 20 and pore size should be carefully evaluated. Brownstein and Tarr proposed three diffusion regime limits, which are assessed based on the rate of molecular transport from the bulk to the surface and how fast relaxation occurs upon contact.^64^eq 20 is only valid in the fast-diffusion regime limit. Under this condition, all fluid molecules have an equal probability of exploring the surface over the experimental time, i.e., diffusion is faster than surface relaxation.^64^ It relies on the assumptions of homogeneous surface chemistry and negligible impacts of diffusional pore coupling or internal field gradients.^62^ In contrast, in systems characterized by the slow- or intermediate-diffusion regime limit, the transport of molecules to the surface is the limiting step, and nuclei relax upon contact.^64^ In this regime, the signal from a single pore is multiexponential, and pore sizes cannot be reliably estimated.^62^

### 1.3 Multiexponential Decay Signals and Relaxation Time Distributions

The use of relaxation time distributions is a suitable approach for the assessment of fluid dynamics in complex porous systems.^40^ Although time-domain NMR cannot resolve functional groups, it can be used to assess nuclei populations with unique relaxation rates. In this case, the magnetization signal is multiexponential and can be represented as a Fredholm equation of the first kind (eq 21):^66^
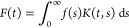
21where *K*(*t*, *s*) is related to a kernel function for *T*_1,2_ and *F*(*t*) and *f*(*s*) are associated with *M*(*t*) and a probability density function of *T*_1,2_, *p*(*T*_1,2_), respectively.^67^ The expression of eq 21 within discrete values, i.e., *j* = 0 to *J*, is determined as eq 22:
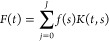
22The equivalent expression in terms of magnetization, experimental time, and relaxation times is expressed in eq 23:
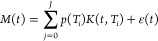
23where ε(*t*) is used to account for experimental noise. Multiexponential time-domain NMR signals are resolved by applying multivariate models, frequently relying on inverse Laplace transforms (ILT).^68^ Such operations are considered ill-posed mathematical problems with nonunique solutions and are addressed by constrained regularizations implemented via computational algorithms.^67^ A valid output relies on important assumptions, where the distribution can only contain positive values restricted within a well-defined range. The chosen constraints are generally determined based on the first acquired echo and the highest relaxation time observable at the testing conditions, i.e., that of the pure bulk fluid.

## 2 TD NMR Applications to Catalysis

### 2.1 Ex Situ Characterization of Catalytic Materials

NMR relaxation represents a valid method for probing surface adsorption and dynamics of molecules over surfaces. In particular, the *T*_1_/*T*_2_ ratio can be considered an indicator for characterizing surface interactions. In recent years there have been several contributions able to provide a correlation between the *T*_1_/*T*_2_ parameter and thermodynamic aspects of adsorption. For example, it has been shown that this ratio can be correlated with an activation energy for surface diffusion, which can in turn be related to the strength of the surface relaxation sinks. This was demonstrated by measuring the *T*_1_/*T*_2_ ratio of water in several porous metal oxides, used as catalyst supports, and comparing such values with the maximum adsorption energy obtained by temperature-programmed desorption (TPD) experiments.^57^ Further work has also shown that a correlation exists between the *T*_1_/*T*_2_ of alcohols adsorbed over porous silica and their adsorption energy as calculated by density functional theory (DFT) calculations.^9^ The behavior of the studied system could be rationalized in terms of hydrogen bonding interactions of the alcohol molecules with the pore surface, in particular, the strongest surface adsorption sites forming multiple bonding interactions with the same adsorbate molecule.

The link between NMR relaxation and surface affinities of adsorbate molecules has important implications in catalysis. Adsorption is an essential step in heterogeneous catalysis. Adsorbate/adsorbent interactions at the fluid/solid interface play a key role as they affect accessibility of reactive species to catalytic active sites.^69^ TD NMR relaxation has, for example, been used to elucidate the role of Au nanoparticle size in the oxidation of glycerol in water. The results indicated that the adsorption properties of glycerol relative to water as a function of the Au loading, measured as *T*_1_/*T*_2_, have a similar trend to that observed for the reactivity, with glycerol exhibiting a higher surface affinity relative to water for the catalyst with smaller Au nanoparticles. This example shows the usefulness of NMR relaxation methods to study surface morphology aspects that are strongly linked to catalytic activity.

**Figure 5:**
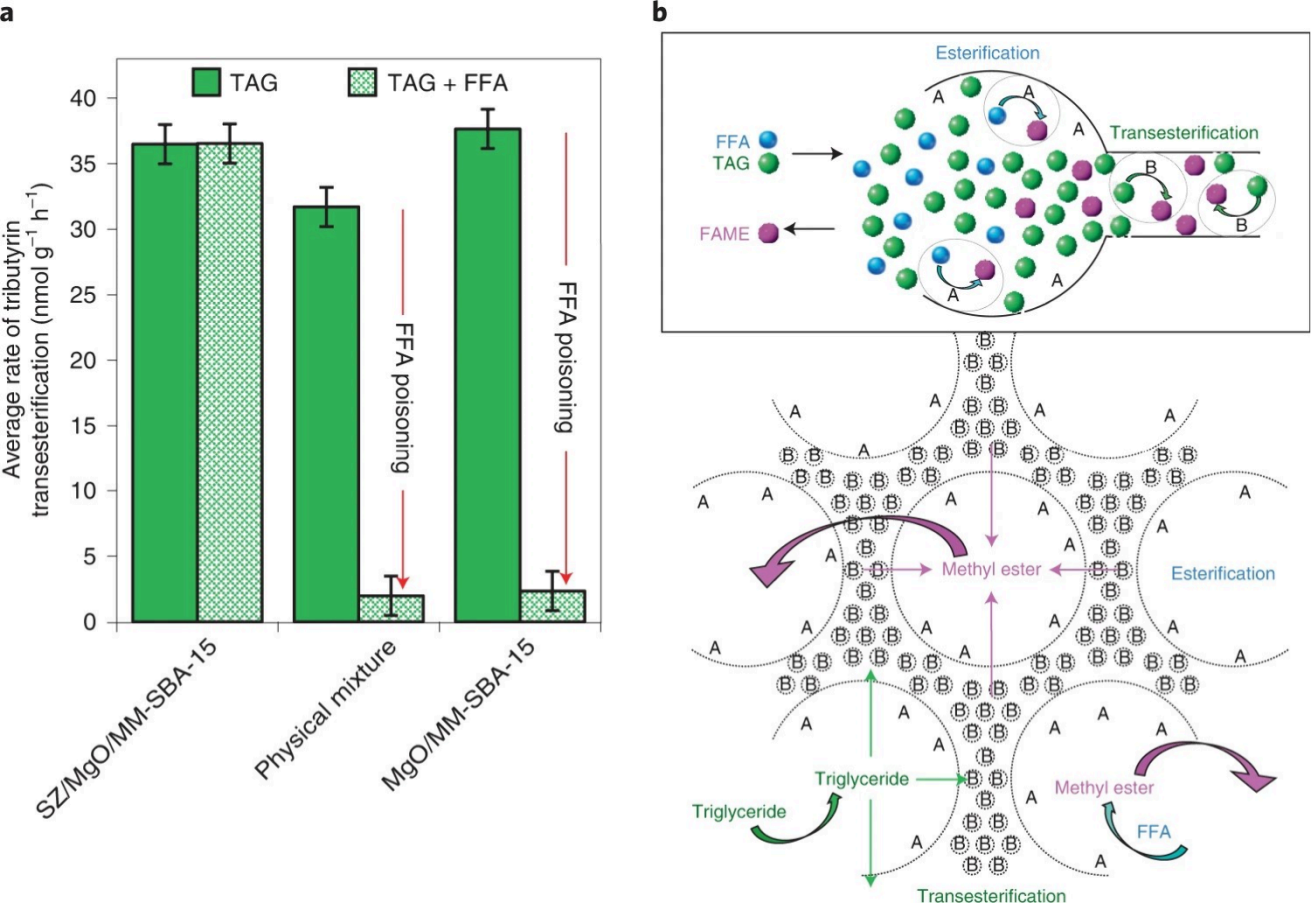
Substrate channeling: esterification and transesterification over acid/base catalysts. (a) Average rate of tributyrin transesterification over SZ/MgO/MM-SBA-15, a 1:1 by weight physical mixture of MgO/MM-SBA-15 and SZ/MM-SBA-15, or MgO/MM-SBA-15 in the absence or presence of hexanoic acid. (b) Schematic of proposed substrate channeling mechanism: (i) TAG+FFA mixture enters macropores; (ii) FFA undergoes esterification over SZ (Acid) sites and is neutralized in macropores; unreacted TAG diffuses and undergoes transesterification over MgO (Base) sites within mesopores. Reproduced with permission from ref (70), Springer Nature.

2D TD *T*_2_*–T*_2_ NMR relaxation-exchange correlation measurements have also been used to elucidate connectivity of different pore environments within hierarchical catalysts used for acid–base cascade and antagonistic reactions.^70^ In the designer materials basic sites are controllably located in mesopores, while acid sites are restricted to macropores, in a manner which limits catalyst poisoning in base catalyzed transesterification (Figure 5).

**Figure 6:**
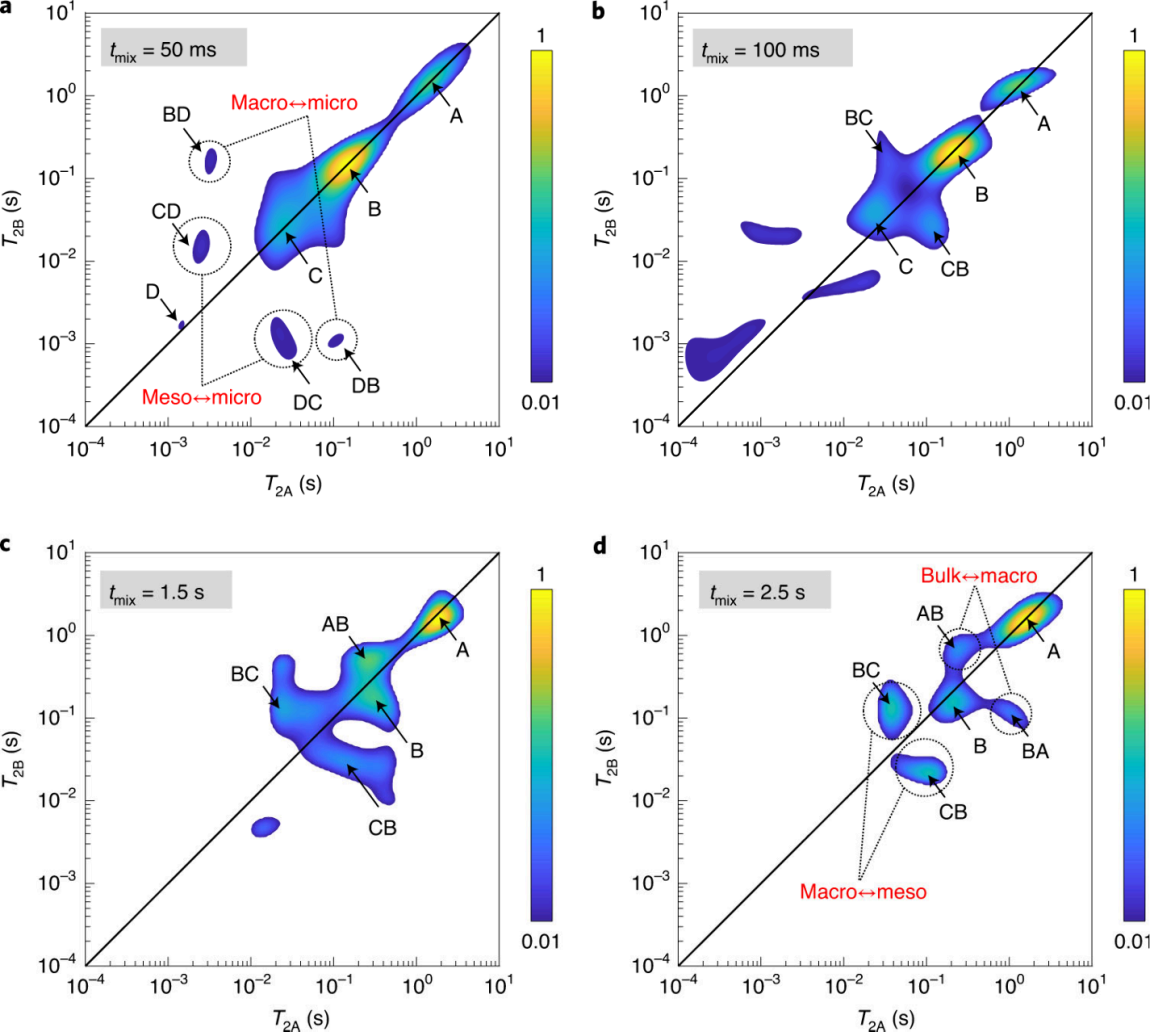
NMR relaxation–exchange correlation data. (a–d) Low-field ^1^H relaxation–exchange correlation plots for water in unfunctionalized MM-SBA-15 with various *t*_mix_ times. Normalized peak intensities are defined by the color bars, which follow a linear scale. On-diagonal peaks A, B, C, and D are assigned to water populations (A) outside the hierarchical pore framework, (B) within macropores, (C) within mesopores, and (D) within micropores, while off-diagonal cross-peaks indicate diffusive exchange between these sites on the time-scale of *t*_mix_. The reduction in peak resolution at short *T*_2_ arises from longitudinal (*T*_1_) relaxation processes during *t*_mix_. Reproduced with permission from ref (70). Copyright 2020 the authors of ref (70), under exclusive license to Springer Nature.

The NMR results were able to reveal key details about pore network connectivity, which would be challenging to elucidate with other techniques. In particular, *T*_2_*–T*_2_ exchange correlation as a function of exchange time (Figure 6) revealed an observable exchange between the macropore population and water outside of the catalyst; conversely, no evidence of exchange between mesopores and water outside of the material was seen, which is crucial to limiting basic site poisoning by free fatty acids (FFA). Specifically, no cross-peaks between A (water between particles) and C (water in mesopores) were observed at any mixing time. These results demonstrated that bulk molecules can only access active sites in the mesopores first going through larger macropores, which catalyze the conversion of FFA poisons to benign fatty acid methyl esters.

**Figure 7:**
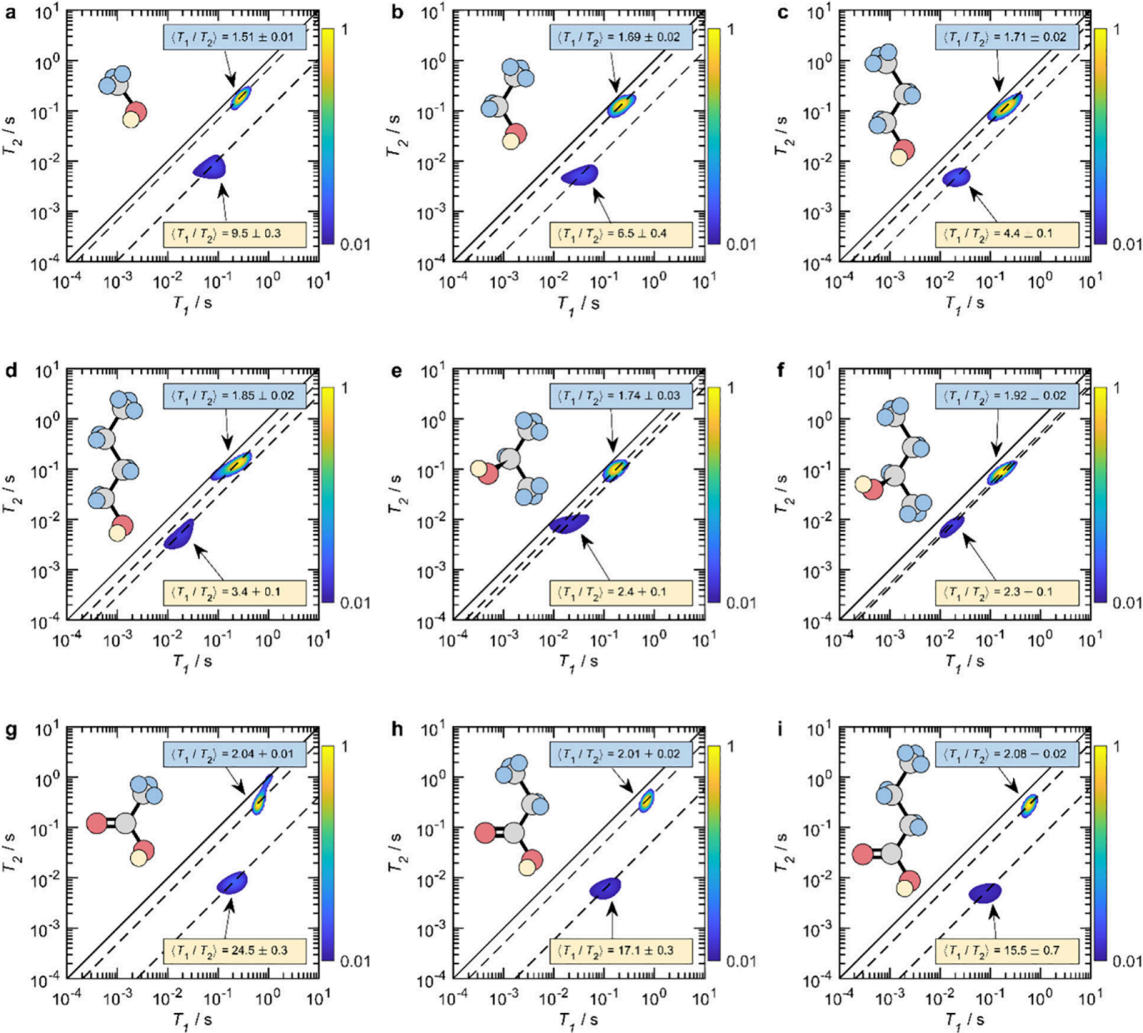
^1^H *T*_1_–*T*_2_ correlation data for (a–d) primary alcohols ((a) methanol, (b) ethanol, (c) 1-propanol, and (d) 1-butanol), (e, f) secondary alcohols ((e) 2-propanol and (f) 2-butanol), and (g–i) carboxylic acids ((g) acetic acid, (h) propanoic acid, and (i) butanoic acid) in mesoporous silica (exhibiting 15 nm pores) at 12.7 MHz. The magnitude of each correlation peak indicates the relative probability of each system exhibiting a particular combination of *T*_1_ and *T*_2_ relaxation times, as indicated by the color bars. Solid diagonal lines indicate the parity ratio *T*_1_/*T*_2_ = 1, while the modal relaxation time ratio ⟨*T*_1_/*T*_2_⟩ of each correlation peak is indicated by dashed diagonal lines; ⟨*T*_1_/*T*_2_⟩ values are specified in each panel. The molecular structure of each adsorbate is also given: C and O atoms are colored gray and red, respectively. Aprotic H are colored blue, while protic H are shown in yellow. Correlation peaks at long and short *T*_2_ are assigned to aprotic and protic ^1^H-containing moieties, respectively. Reproduced from ref (71). Copyright 2021 American Chemical Society.

Despite the lack of spectral resolution, TD NMR relaxation can distinguish functional group-specific relaxation phenomena of molecules confined in porous catalytic materials. A notable example is a recent work of Robinson et al.,^71^ who used 2D *T*_1_*–T*_2_ NMR relaxation to show functional group-specific nuclear spin relaxation phenomena associated with the alkyl and hydroxyl ^1^H-bearing moieties of alcohols and carboxylic acids adsorbed in mesoporous silica. The relaxation characteristics of these groups could be clearly resolved in the *T*_1_*–T*_2_ distribution maps, as shown in Figure 7. The cross peaks with lower *T*_1_ and *T*_2_ correspond to O–H species with greater interaction with the surface compared to alkyl C–H species. Another interesting finding of their work was a clear link between the difference in *T*_1_/*T*_2_ ratio of the hydroxyl and alkyl moieties of the adsorbed species, that is, (*T*_1_/*T*_2_)_hydroxyl_ – (*T*_1_/*T*_2_)_alkyl_, and the acidity of the adsorbate.

Glucose-based chemistry has gained significant attention in recent years, due to the need to enable a more sustainable chemistry based on renewable feedstocks. Glucose is a versatile renewable feedstocks, which can be converted to a variety of chemical commodities, such as ethanol, lactic acid, furfural and 5-hydroxylmethylfurfural, the latter being of significant interest.^72^ Such reactions are usually carried out in liquid-phase, in the presence of a solvent, typically water, using zeolites as solid catalysts. These systems are well suited to NMR relaxation studies, which can give important insights into fluid/solid interactions affecting catalyst performances. In this context, it has very recently been shown that the *T*_1_/*T*_2_ ratio of pyridine in HZSM5 zeolites exhibit strong sensitivity to the silica/alumina ratio (SAR) of these zeolites, which is indicative of material acidity.^73^

**Figure 8:**
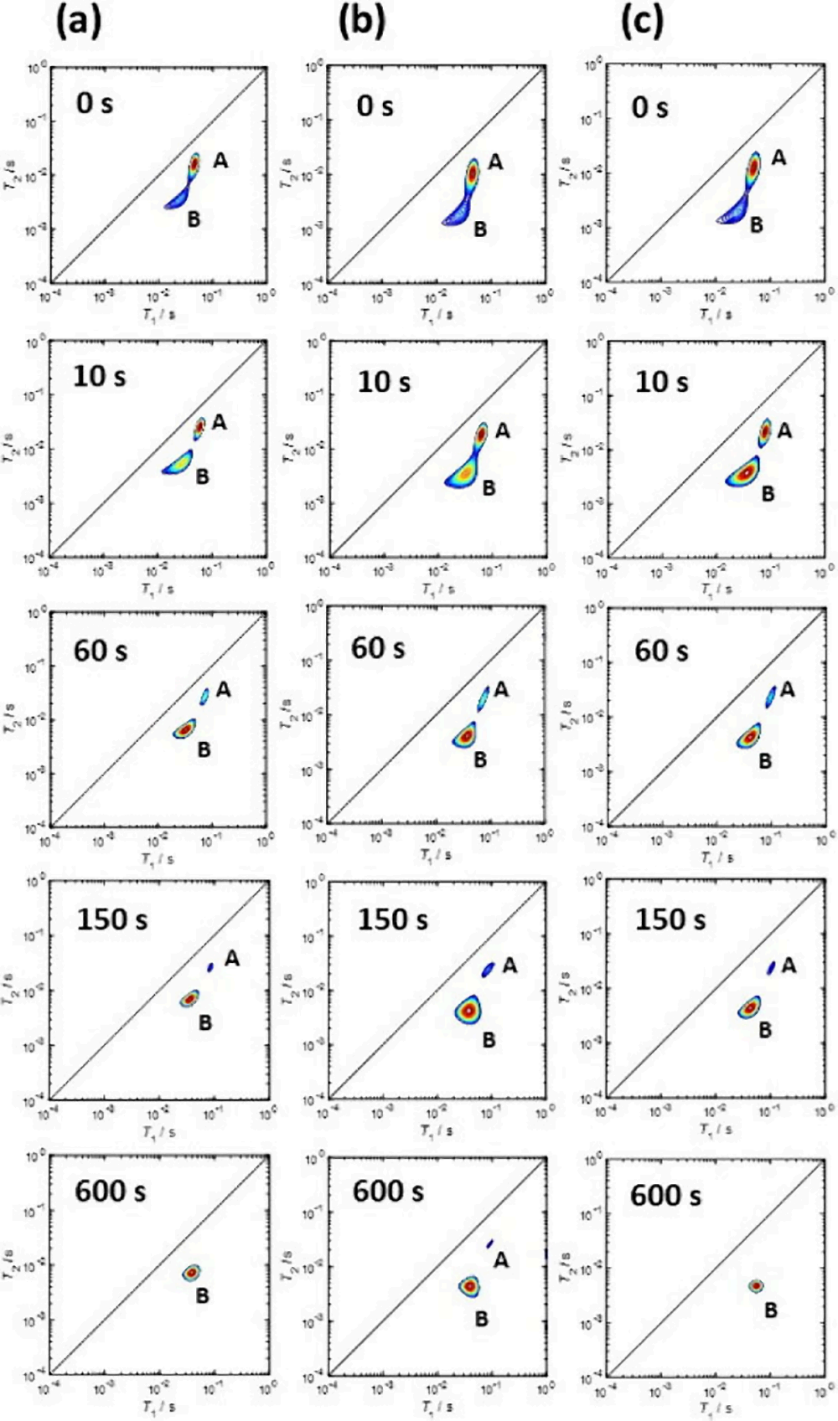
*T*_1_–*T*_2_ relaxation correlation plots for methanol being displaced by water within the pores of (a) HY, (b) Ga/Y and (c) Sn/Y at the displacement time intervals of 0, 10, 60, 150, and 600 s. A and B represent the aliphatic peak of the alcohol (methanol) and the OH/water peak, respectively. Reproduced from ref (74). CC BY 4.0.

The use of such measurements can be very useful to gain insights into catalytic activity of zeolites used in glucose-based reactions. For example, Forster at al. have used *T*_1_*–T*_2_ displacement experiments of water/alcohol mixtures in zeolite Y to study the affinity of these solvents for the zeolite surface.^74^ The results suggested that the lack of catalytic activity of zeolite Y for the isomerization of glucose to fructose in water can be attributed to the strong adsorption of this solvent within the zeolite pores blocking reactants from the Lewis acid sites active for the sugar isomerization. Water was also seen to be able to completely displace methanol from the zeolite pore space, as evidenced by the *T*_1_*–T*_2_ maps showing the complete disappearance of the methanol peak, being displaced by water, after approximately 600 s, as shown in Figure 8.

**Figure 9:**
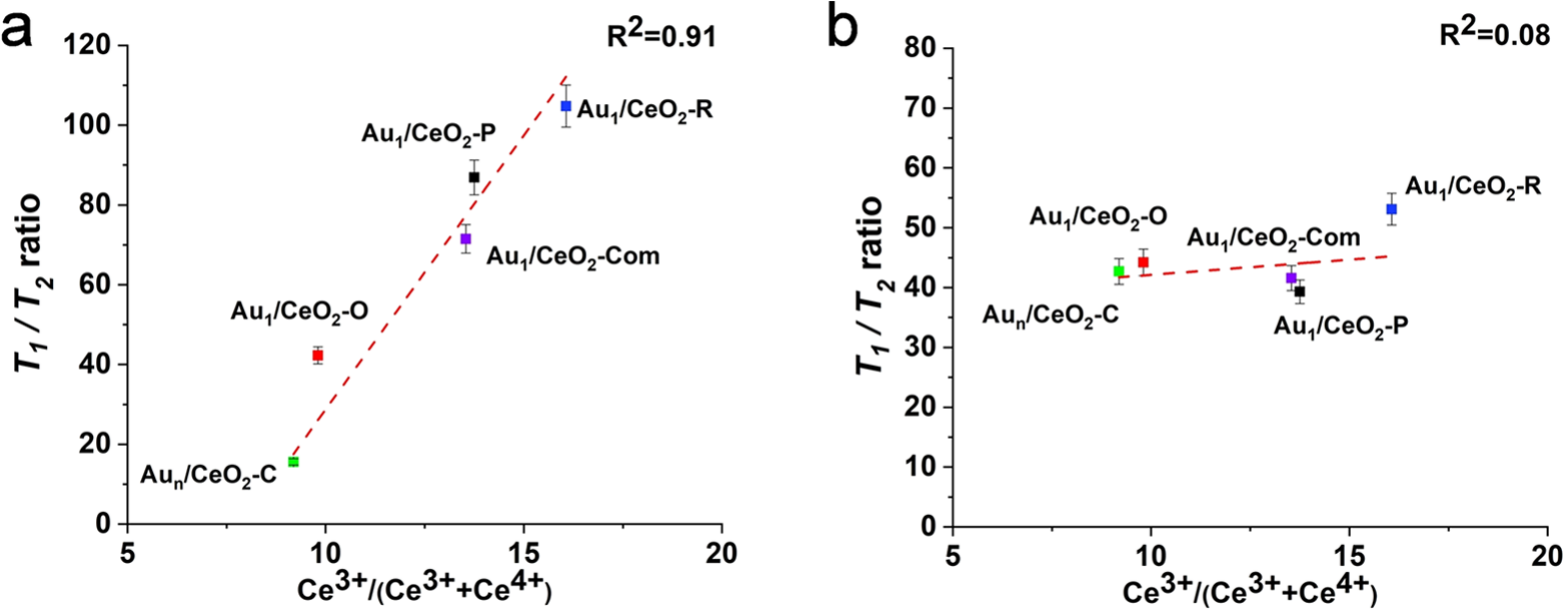
Correlation between *T*_1_/*T*_2_ ratios and Ce^3+^ content for (a) benzyl alcohol and (b) benzaldehyde adsorption on Au_1_/CeO_2_-X and Au_*n*_/CeO_2_-C catalytic materials. Reproduced from ref (77). CC BY 4.0.

Espinat and co-workers have examined asphaltene adsorption in alumina catalyst pores using *T*_1_/*T*_2_ NMR readily differentiating the probe molecules’ restricted diffusion relative to the more mobile solvent molecules.^75^ Another study by Dimitratos and co-workers on preparation of Au colloidal nanoparticles for glucose oxidation reported surface affinity of reaction products, as measured by *T*_1_/*T*_2_ NMR, can affect catalyst deactivation.^76^ In particular, it was suggested that the strong adsorption of glucaric acid, which is the oxidation product of the gluconic acid intermediates, inhibits further conversion of gluconic acid. In a very recent work, NMR relaxation measurements have revealed some peculiar insights into the impact of ceria support morphology on Au single-atom catalysts used for the benzyl alcohol selective oxidation.^77^ In particular, a strong correlation between the *T*_1_/*T*_2_ ratio of benzyl alcohol and the content of Ce^3+^ ions was observed (Figure 9). This experimental finding is in agreement with computational studies showing that Ce^3+^ is crucial for the benzyl alcohol oxidation as it acts as the main adsorption site for alcohol adsorption.^78^ Conversely, no correlation was observed for the benzaldehyde product (Figure 9), which rules out competitive adsorption of the product.

**Figure 10:**
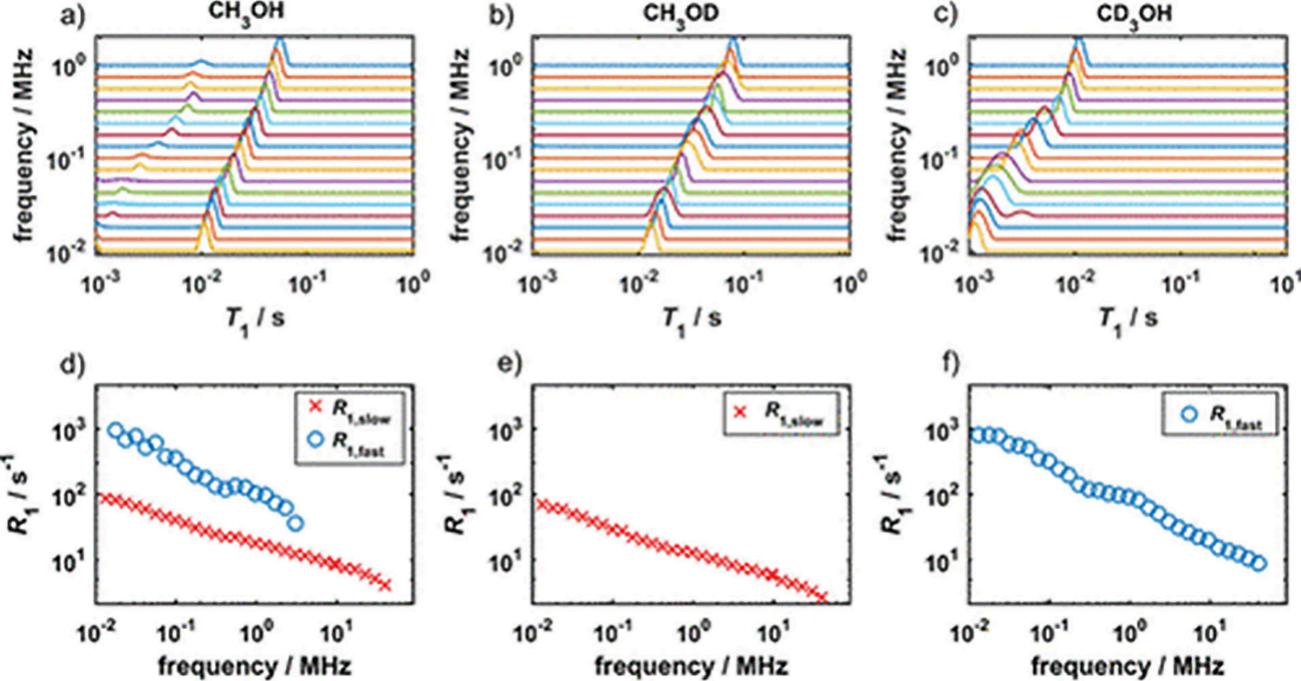
Normalized *T*_1_ distributions, recorded at a frequency value of ≤1 MHz and, below, the corresponding NMRD profile calculated from the modal values of the *T*_1_ distributions for (a, d) CH_3_OH, (b, e) CH_3_OD, and (c, f) CD_3_OH. The estimate of the relative population of the minor peak is calculated from data in the frequency range 0.1–1.0 MHz. Reproduced from ref (79). Copyright 2018 American Chemical Society.

Applications of FFC NMR to study catalytic materials have been recently reported and they usually aim at unravelling adsorption behavior and molecular dynamics of molecules adsorbed over catalyst surfaces. Gladden and co-workers reported FFC NMR studies of protic and aprotic solvents on γ-alumina surfaces of catalytic interest.^79^ The data showed the presence of two distinct peaks in the *T*_1_ distribution of both methanol and acetone. The two peaks for methanol were assigned to the presence of two different chemical interactions with the surface, the hydroxyl and alkyl moieties of the molecule. This was also demonstrated by using partially deuterated methanol species, CD_3_OH and CH_3_OH, imbibed in the alumina support (Figure 10), which showed that the two-component behavior of methanol in γ-alumina is reduced to a single-component both CD_3_OH and CH_3_OD, coming, respectively, from a fast relaxing minor environment, associated to hydroxyl protons, and a slow relaxing major environment associated with the alkyl protons. In the case of adsorbed acetone, the second environment was assigned to a stable reaction intermediate formed during an aldol reaction. The same method has also been used to study the behavior of binary mixtures over γ-alumina support. Two binary systems were studied, cyclohexane/THF and methanol/THF. For both systems, changes in relaxation behavior with composition were ascribed to changes in surface accessibility of each species, which is related to microphase separation at the pore surface. In particular, a key conclusion of this study was that the more polar species in the mixture exists in a surface layer phase rich in that species relative to the overall composition inside the pore space. This highlights the usefulness of NMR methods in characterizing surface compositions, which is of great importance in catalytic science.

NMR methods are also able to directly access diffusion coefficients on probe molecules confined in porous catalysts, hence they yield important information on the transport-structure relationship in heterogeneous catalysis. It is noted here that most NMR diffusion studies are based on pulsed-field gradient (PFG) Fourier transform-based NMR spectroscopy methods.^80,81^ Such methods have been widely used to probe diffusion in catalytic materials, including mesoporous oxides,^82−85^ activated carbons,^86^ and zeolites.^31,87−89^ Other approaches, including Laplace NMR diffusion experiments,^90,91^ and the alternating pulsed field gradient stimulated spin echo (APGSTE) have also been reported.^92^ NMR methods are well-suited for studying mechanisms of diffusion. In the Rouse mechanism^93^ molecular segments displace in a lateral direction into pockets of free volume facilitating motion of the full molecule, while the Zimm mechanism^94^ extends the Rouse model acknowledging that segment motion will lead to displacement of neighboring chains within a hydrodynamic-coupling length scale. Other mechanisms have been studied and broadly labeled reptation mechanisms, which acknowledge resistance to lateral motions associated with either chain entanglement or in the case of porous media, restricted diffusion under confinement. In reptation mechanisms motion is limited to creeping motions in the longitudinal (pore) direction.^92^ The APGSTE method has recently been employed as a powerful tool for resolving these mechanisms of diffusion for alkanes in the pores of catalysts^92^ and future applications are expected to provide insights in liquid phase catalysis.

Other applications of TD NMR methods have also successfully been used to probe diffusion of fluids in porous catalysts. Belén Franzoni and co-workers used *T*_1_/*T*_2_ relaxation maps to study mesoporous silica saturated with linear and cycloalkanes.^95^ The authors showed that both pore size and intermolecular interactions influence fluid dynamics characterization via TD NMR. For instance, the regressed longitudinal and transverse surface relaxivities were notably higher for *T*_1_ and *T*_2_ peaks attributed to fluid molecules confined in mesopores <6 nm. Another notable work was recently published by Gladden and co-workers, who carried out low-field TD PFG NMR measurements to characterize the tortuosity (calculated as the ratio of the bulk self-diffusivity of a fluid to the self-diffusivity of the same fluid inside a porous structure) of catalytic materials with a high content of paramagnetic species.^30^ The method is a notable application of low-field NMR, through which the perturbations of the paramagnetic component are limited by field strength, i.e. such measurements are not feasible using conventional high field methods. In particular, tortuosity measurements of TiO_2_ supports containing 1 wt.% of manganese oxide (MnO_2_) as well as TiO_2_-supported cobalt oxide (Co_3_O_4_) catalysts with a 20 wt.% of cobalt, could be obtained. The results showed that the effect of cobalt deposition on the tortuosity of the porous structure was significant and is influenced by catalyst preparation method.

### 2.2 Characterizing Surface Area and Porosity

**Figure 11:**
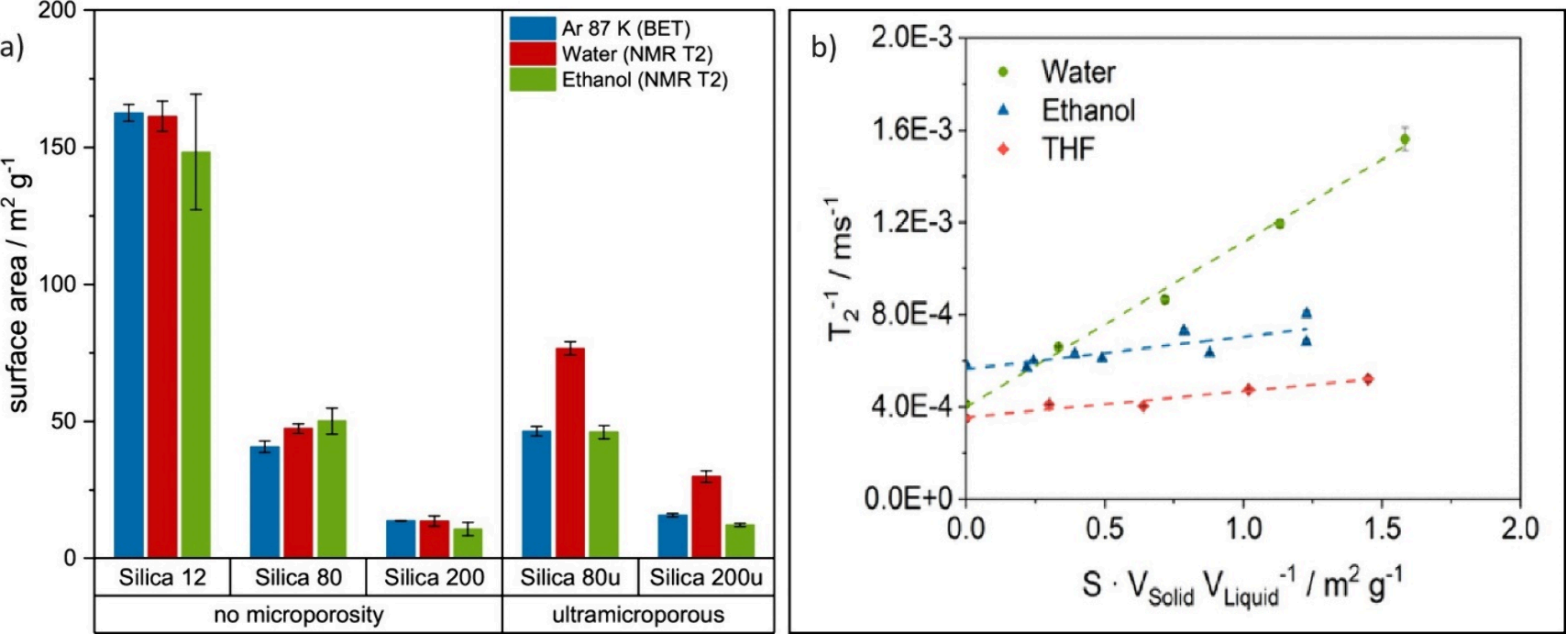
(a) Specific surface area of nonmicroporous Silica 12, Silica 80, and Silica 200 as well as ultramicroporous Silica 80u and Silica 200u determined with Ar 87 K adsorption and NMR *T*_2_ in water and ethanol (reference system: silica in water and silica in ethanol). (b) Comparison of *T*_2_ calibration lines of Silica 200 immersed in water, ethanol, and THF. Reproduced from ref (10). Copyright 2023 American Chemical Society.

As discussed in Nuclear Magnetic Resonance Fundamentals, Brownstein and Tarr recognized that in the fast diffusion regime, the NMR relaxation rate in low-strength magnetic fields correlates to the surface area to volume ratio of a sample or mode of pore volume distribution (applies to both spin–lattice and spin–spin relaxation rates, e.g., eq 20).^64^ The physical phenomenon has been applied with great economic impact in the fields of geology and petroleum engineering,^63^ but synthetic materials have offered a platform for rational development of these correlations. Smith and co-workers provided early demonstrations correlating spin–lattice relaxation rates (1/*T*_1_) at low-fields to wetted porosity in controlled, synthetic silica materials, including calculating distributions of *T*_1_ from multiexponential decay data.^8,96−100^ The technique was readily extended to spin–spin relaxation rates (1/*T*_2_) and fluids other than water.^101^ Renewed attention in the characterization of nanostructured carbons^102^ and silica supports^10,103^ may be leveraged in the characterization of catalyst supports. Gallego-Gómez et al. demonstrated the capability of TD NMR in a benchtop, low-field instrument to characterize microporosity in Stöber silica particles.^104^ Thommes and co-workers provide quantitative comparisons of argon adsorption and TD NMR surface areas for various silica particles, demonstrating that the choice of imbibed/adsorbed fluid impacts the measurement. Water (by TD NMR) and carbon dioxide (by gas adsorption) with their low kinetic diameters reveal ultramicroporosity not apparent in Ar adsorption (Figure 11a).^10^ The same study demonstrated that the sensitivity of TD NMR data is a function of the fluid-surface interaction energy, consistent with trends in *e*_surf_.^57,103^ As shown in Figure 11b, water is a more sensitive probe of wetted surface area than either ethanol or tetrahydrofuran for silica.^10^ Given the calibration curve of Figure 11b, the surface area of future samples can be conveniently correlated to the spin–spin relaxation rate (1/*T*_2_). In a complementary effort, Suekuni and Allgeier demonstrated that the surface chemistry of a given particle significantly influences the surface relaxivity (eq 20) and, hence, the sensitivity of TD NMR for determining wetted surface area for a given surface/fluid pair.^41^ A correlation between particle surface chemistry attributes and surface relaxivity has been elucidated, allowing computation of surface relaxivity for unknown samples and laying a foundation for generating larger corroborating data sets.^41^ Paramagnetic impurities can strongly influence the calibration of TD NMR surface areas. Under multiple conditions this influence has been modeled as a linear dependence allowing regression of surface relaxivity in the absence of paramagnetic impurities and more importantly increasing the accuracy of TD NMR for samples unavoidably influenced by paramagnetic impurities.^37,45,62^ While complementary to gas adsorption methods for determining specific surface area, it is notable that TD NMR methods allow data collection in a fraction of the time required for gas adsorption, e.g., data acquisition may be less than 4 min for thermally equilibrated samples.

### 2.3 In Situ/Operando TD NMR Studies

Critical to all operando TD NMR studies is availability of suitable reaction cells. The requirement for nonmetallic cells, held in the magnetic field of an NMR, in a bore spanning greater than 50 cm length is unique to NMR compared to various other operando characterization techniques.^105^ These material and format challenges, coupled with the need of many catalytic reactions to access metallic active sites, and high temperatures and pressures, necessitates specialized equipment in stopped flow,^106^ traditional bore,^107^ solid-state, magic-angle spinning^108^ or novel low-field^109^ instrumentation. Collectively these requirements increase capital investment and restrict access of the methods to a relatively small number of specialized groups.^6,12,56,79,107,108,110−117^ However, the developments in commercially available, low-field, permanent magnet instruments over the past decade have increased access to operando NMR methodologies employing time-domain and spectroscopic techniques.^8,98,114,116,118−123^

**Figure 12:**
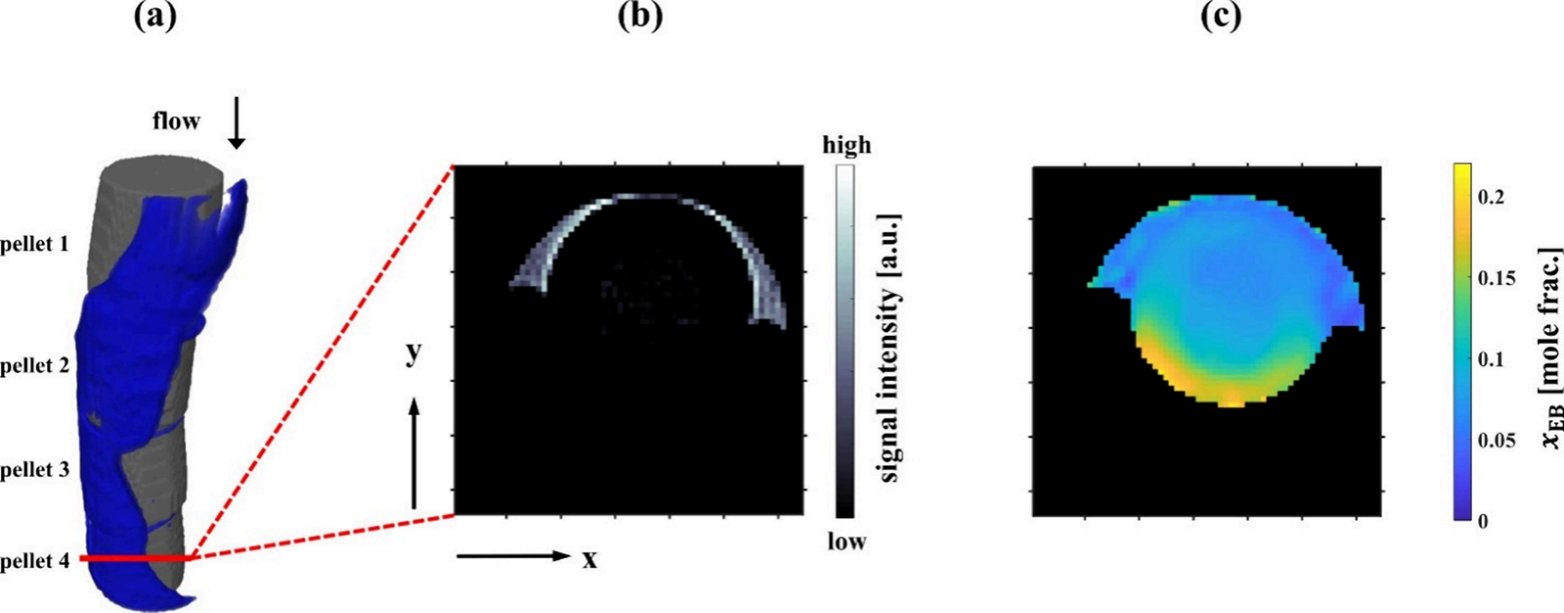
Reactor structure, liquid distribution, and resulting composition map for a fixed bed reactor employed for styrene hydrogenation using Pd/Al_2_O_3_. (a) Reactor structure and interpellet liquid as measured using 3D RARE MRI. Gray shading represents catalyst pellets and blue shading represents liquid flowing over the catalyst. (b) 2D slice of 3D ^1^H signal intensity image acquired using RARE MRI at TOS = 30 h. (c) Image of ethylbenzene concentration, *x*_EB_, at TOS = 38 h as calculated from 2D MRSI. Images shown have a FOV of 5.5 mm (*x*) × 5.5 mm (*y*) and an isotropic in-plane spatial resolution of 86 μm. The slice thickness is 2 mm for the composition map shown in (c), while the image shown in (b) has a resolution of 86 μm in the direction into the page. Reproduced with permission from ref (12). Copyright 2023 Elsevier..

In the realm of high-field NMR, Gladden, Mantle, and co-workers have recently developed methods for characterizing maldistribution of liquid in trickle bed reactors using operando NMR imaging.^12^ While many operando spectroscopy techniques probe phenomena at the molecular scale (Å to nm), NMR imaging is particularly suited to characterize phenomena relevant to transport on scales of μm to cm. The technique, Rapid Acquisition by Relaxation Enhancement (RARE), to visualize a liquid full, packed bed of catalyst particles differentiated interparticle from intraparticle fluid.^12^ Imaging contrast was afforded because fluid inside the pores had significantly lower *T*_2_ than fluid between particles. This same study of styrene hydrogenation over particulate Pd/Al_2_O_3_ was elaborated using three-dimensional magnetic resonance spectroscopic imaging (MRSI) (Figure 12). The combination of time-domain and spectroscopic data enabled characterization of maldistribution in the wetting of the catalyst particles. Intriguingly, greater conversion to ethylbenzene was observed in the gas enriched portion of a single particle compared to the liquid-dominated regions an insight unattainable by any other technique, but consistent with predictions based on finite element computational modeling.^124^

Such studies applying magnetic resonance imaging of fixed bed catalytic reactions emerged from foundational work in the early 2000s^125,126^ and general magnetic resonance imaging techniques developed even earlier^127−130^ and have been reviewed by Pesch.^6^ Regarding precedent, Koptyug and co-workers provided seminal exploration of three phase hydrogenation using MRSI and demonstrated the value of a packed bed reactor in a high-field NMR instrument to characterize imperfect fluid flow and reaction profiles in a three-phase reactor.^131,132^ Similar techniques have been applied to gas phase hydrogenation of ethylene, even though the high diffusivity presents a practical challenge to imaging techniques.^133^ The investigators hypothesized that saturation effects led to artifacts under-estimating the population of ethane product. The use of 30° excitation pulses, increased spacing of observed slices in the direction of flow and characterization of long *T*_1_ values were employed to ameliorate the effects of these artifacts.^133^

**Figure 13:**
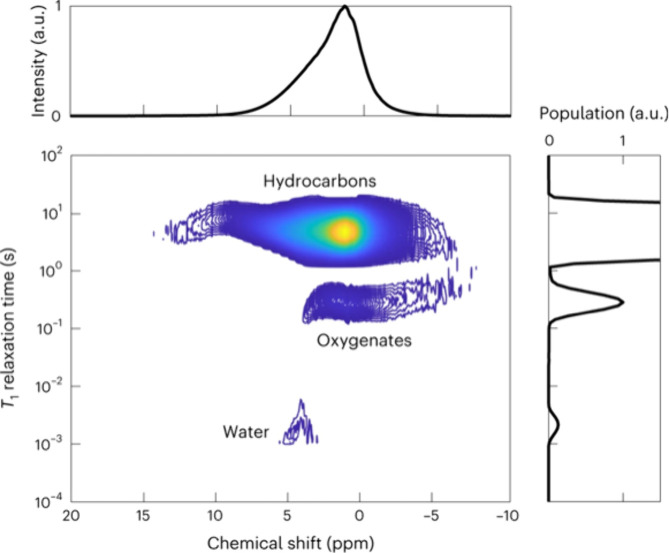
Operando characterization of water in catalyst pores. The 2D ^1^H spectrum, correlating chemical shift and *T*_1_ relaxation time, is used to discriminate hydrocarbons, oxygenates and water present in the reactor. Reproduced with permission from ref (107). Copyright 2023 the authors of ref (107), under exclusive license to Springer Nature.

Fischer–Tropsch synthesis has been characterized using a combination of operando TD NMR and MRSI in a seminal study that demonstrated differences in the bulk averaged product molecular weight distribution by gas chromatography and the in-pore molecular weight distribution determined by NMR.^107^ Using an advanced pulse sequence, correlations between chemical shift and relaxation time, furthermore, definitively characterized a water rich layer at the catalyst surface (Figure 13). Note that the *T*_1_ value of the water peak is low indicating surface confinement.

The development of permanent-magnet, benchtop instruments of moderate fields (e.g., 60 MHz for ^1^H) facilitates laboratory-based operando NMR characterization of heterogeneous catalysts employing both spectroscopy and a variety of low-field TD NMR techniques. Demonstrative of the potential from this integrated approach, the hydrogenation 1-octene was evaluated over a Pd/TiO_2_ catalyst. While spectral resolution is reduced in benchtop NMR, conversion was readily obtained by identification of well-resolved resonances. Furthermore, the relative adsorption strength of the starting material and product were characterized by *T*_1_/*T*_2_ 2D data sets that differentiate the alkane product from the alkene starting material and the use of PFG enabled the determination of the restricted diffusion of fluids in the pore system, notably differentiating alkene with about 10% lower diffusivity in accord with the stronger interaction with the surface.^114^ Such PFG probes have also been deployed to characterize intracrystalline zeolite diffusivity of small molecules in the presence of dominating interparticle diffusivity.^31^ Correlations between fluid *D* and *T*_2_ in an operando TD NMR probe comprising a trickle bed hydrogenation reactor resolved the extent of reaction at different positions in a packed bed reactor.^115^ Such spatial resolution is unattainable by other methods of operando spectroscopy with a single detector.

### 2.4 Notes on Scientific Rigor in TD NMR

It is clear from the discussion above that TD NMR is a very useful tool for unravelling aspects related to surface dynamics, diffusion and adsorption within the catalyst structure, and it complements more conventional catalyst screening and characterization methods. With an interest in improving quality in scientific research, authors have recently focused on documenting recommendations for achieving rigor and quality in catalysis experiments,^134−136^ which prompts us to provide a similar perspective on TD NMR. Given the breadth of applications under the umbrella of TD NMR, it is infeasible to define standard, control samples and experimental practices for all applications.

TD NMR specific surface areas and pore size distributions do offer a sufficiently narrow scope for defining control samples and practices. Indeed, for gas physisorption, for example by the BET specific surface area method,^137^ the International Union for Pure and Applied Chemistry sanctioned an important working group to establish standards.^138^ We recommend that the community establish a similar working group for TD NMR methods. Among the most important considerations for attaining reproducible surface areas are a baseline characterization to assess if paramagnetic centers could be influencing spin–spin relaxation,^10,37,62,139^ and evaluating if the particle system under analysis is truly in the fast-diffusion regime defined by Brownstein and Tarr.^64^ For the latter we recommend characterization of samples at different ratios of solid to fluid to assess goodness of fit for linearity in eq 20. The absence of a good fit may imply that pores in the sample hold fluid that is not in fast exchange with the fluid outside the pore. Additionally, experimental data should be subject to the inverse Laplace transform to assess if pore fluids are easily resolved, consistent with eq 23.^68^ The presence of a significant multimodal population requires a more complicated assessment of wetted surface area and prompts analysis of the data as pore volume distribution. Here it is notable that both Gallego-Gómez et al.^104^ and Schlumberger et al.^10^ characterized Stöber silica particles. The latter study focused on characterizing stable aqueous suspensions of the particles (e.g., <10% w/w silica), yielding TD NMR surface area, while the former focused on characterizing well-packed colloidal crystals of settled particles in a regular array (e.g., >75% w/w silica) and yielding pore volume distributions. The studies are differentiated and complementary. The analysis of particle suspensions should always be accompanied by an assessment of settling on the time scale of the data acquisition, as significant settling would lead to multimodal distributions of relaxation times, complicating determination of surface areas.

TD NMR for fluid adsorption strength. As with surface area determinations, rigor in sample preparation is critical in other catalyst characterization methods by TD NMR. When assessing fluid adsorption strength by *T*_1_/*T*_2_ or *e*_surf_ it is critical to control the surface chemical state before exposure to imbibing fluids. This can be particularly challenging for silica surfaces with a high affinity for adsorbed water in laboratory environments, and even more so when microporosity is present.^57,73,140^ Furthermore, high temperature treatments may affect the surface chemistry/degree of hydroxylation or alkoxylation of silica surfaces and researchers are advised to critically evaluate literature acknowledging that different studies may have different catalyst pretreatment methods. The choice of whether to characterize particle suspensions or macroscopic wetted particulates (e.g., in the mm size range) is also notable. Relaxation times are dependent upon the frequency of fluid surface interactions and hence incumbent in the analysis of particle suspensions is the challenge to control the uniform spacing of particles. We recommend the analysis of macroscopic wetted particles, e.g. formed catalyst extrudates, tablets or sphere, fully imbibed with fluid but with negligible extra-particulate fluids to facilitate reliable data analysis.^9,57,141^ Maintaining a vapor saturated atmosphere above the wetted particles is another critical-to-quality practice to minimize fluid evaporation during the measurements. This can be achieved by placing a wetted fabric in the headspace above the sample but out of the NMR coils. This experimental technique also facilitates characterization of intrapore fluid diffusivity (constrained diffusion), though other options are available to characterize catalyst beds comprising both intraparticle and interparticle fluids.^12,31,107,115^

Data Analysis. Many TD NMR methods require fitting of experimental data to either a monoexponential function or regularization and inversion of the data to reveal multiple populations; these may include analysis by the inverse Laplace transform.^66,68,142−144^ Acknowledging that ILT is an ill-posed problem with no unique solution, all numerical methods must have constraints and lack of uniformity across laboratories may be particularly problematic, prompting an assessment of reproducibility. Here we also recommend that a working group consider opportunities for ensuring greater uniformity. In the interim we advise researchers to clearly disclose numerical methods utilized in their studies and, most importantly, to maintain uniformity of these methods so that at minimum, a study of multiple samples is conducted with a common basis.

## 3 Opportunities for Growth in TD NMR

Given the diverse applications for TD NMR and the relative uptake of the methods compared to widely accessible tools like physisorption, chemisorption, and temperature programmed desorption, we see great potential for growth in this field. Table 1 of this review provides a summary of the utility of various TD NMR methods, and we complement it with Table 2, placing the utility of TD NMR in the context of various other methods of characterization in catalysis. While any such effort must be an oversimplification, the table demonstrates that as a family of methods, TD NMR is uniquely suited to characterize the distribution, movement, and interaction of fluids with surfaces and through catalyst pores and complements other methods of characterization. TD NMR methods are not well-suited to some critical aspects of catalysis, notably the measurement of individual component concentrations that enable kinetic modeling and molecular-scale information about the evolution of active sites. TD NMR methods are complementary to techniques like X-ray diffraction (XRD), X-ray absorption spectroscopy, including extended X-ray absorption fine structure (EXAFS), vibrational spectroscopy, and electronic spectroscopy, which all are well-suited to characterizing molecular scale phenomena relevant to the chemical state of catalyst active sites. Furthermore, a number of standing challenges within the field of TD NMR remain, including (1) limited resolution and sensitivity, (2) dependence upon mathematical inversion of data, (3) complicated analysis of materials bearing ferromagnetic components (e.g., Fe, Ni, and Co), which may be essential or inherent in catalysis, and (4) the role of background magnetic field gradients in perturbing intrinsic *T*_2_ values.

**Table 2:** Comparison of Characterization Methods

Methods	Concentration Profiles/Kinetics	Porosity/Coking	Adsorption Strength	Component Diffusion	Active Site Evolution	Multiscale Fluid Distribution
TD NMR		√	√	√		√
Spectroscopy (NMR, vibrational, etc)	√				√	
Gas Adsorption		√				
TPD/TPR/TPO		√	√		√	
Tracer Study				√		
Others (UV–vis, XRD, XAS, XRF, ICP)	√				√	

The limited resolution and sensitivity of NMR are inherent in the physics (see eq 3) and while NMR spectroscopy sensitivity is improved by utilizing higher field strength, many TD NMR techniques are reliant upon low field strength (Section 1.2.2) to accurately infer porosity and adsorption strength. Addressing these inherent limitations, improvements in electronics for radiofrequency pulsing and signal detection have enabled a new generation of low-field instruments with improved resolution and sensitivity compared to older technologies. It is additionally noted that a limitation in TD NMR is reliance upon numerical methods for inversion of the time-domain raw relaxation data to a spectrum of relaxation times. Such distributions may be quantitatively interpreted as discussed in preceding sections, but all result from the inverse Laplace transform, a mathematically ill-posed problem with no unique solution and, in some, variants requiring advanced knowledge of the signal-to-noise ratio for each data set and a reasonable starting estimate of the number of subpopulations. These conditions may present a challenge for cross-laboratory validations. Recent advancements apply statistical learning tools to relieve reliance on advanced knowledge of the solution^145^ and apply alternate regularization constraints to improve the quality of the output.^146^ Beyond mathematical optimization strategies, we encourage the community to prepare control samples for validation of new numerical methods against known pore volume and surface relaxivity parameters.

The characterization of catalyst samples bearing ferromagnetic components such as Fe, Ni, and Co presents the complication that the rate of NMR relaxation is accelerated for fluids in proximity of such components. Particularly in cases where the components are not uniformly distributed, such acceleration (e.g., low *T*_2_ values) could be misinterpreted as smaller pores or a broader distribution of pores than in the absence of such components. Such perturbations are limited at low-field strengths but must be acknowledged when drawing conclusions. A directly related problem arises from any source of magnetic susceptibility gradients for fluids in contact with a surface. Low coordination sites, surface imperfections, and paramagnetic centers may result in local perturbations in relaxation rates. Analyses should be conducted in the fast diffusion regime (Section 1.2.3) with low echo time separation to limit such influences. Mitchell et al. introduced a method to extract intrinsic relaxation times even from higher field instruments operating with samples inducing internal field gradients.^147^

Even in light of the above challenges, the rapidly emerging access to low-cost, low-field, benchtop NMRs, which are especially suitable to TD NMR studies will assuredly widen access.^119^ Conversely, some methods require highly specialized cells and instrumentation but even here the potential of enhanced imaging unattainable by other methods^12,107^ is sure to spur investment by a greater number of geographically dispersed experts. Whether through high-field NMR applied to assessing fluid distribution in process development-scale, trickle-bed reactors or through FFC instruments for assessing surface dynamics, specialized equipment provides exciting opportunities. We particularly see potential in the field of characterizing gas-expanded liquids, which offer notable enhancements of solute diffusivity,^148,149^ and may impact fluid distribution in packed beds, catalytic rate and reaction selectivity. Notable applications to electrocatalysis,^150^ particularly for carbon dioxide reduction are likely to emerge. Further, we see profound opportunities for collaboration between experts in computation (both DFT and molecular dynamics) and those conducting TD NMR investigations.

With increased attention on heterogeneous catalysis conducted in liquid phase, solvent screening could benefit significantly from NMR relaxation measurements, which are able to characterize surface affinities of solvents over different catalytic surfaces with relatively fast experimental times, which can be down to a few minutes. The method could be useful both as a prescreening tool for solvent selection and for elucidating mechanistic influences of solvent upon reaction selectivity. Engineered catalyst materials with controlled wettability^151^ comprise a further area of growth for characterizing molecule dynamics and adsorption strengths as a function of surface chemistry modification. Further insights into surface dynamics can be obtained by performing NMR relaxation measurements at different field strengths, as is the case in FFC NMR. The modeling of such data, although often challenging, is able to yield information on important surface parameters, such as surface diffusion time and surface residence time,^152^ which are important when considering molecular species reacting on solid surfaces.

Catalysis in condensed phase has spurred synthesis of hierarchical materials to facilitate transport.^153−155^ Diffusion NMR methods offer a tool to quantitatively assess the changes in diffusion inside catalyst structures due to the introduction of macro/meso/micropores, and facilitate mass transport in such materials, which is particularly important when dealing with reactions involving components with large kinetic diameter.^156^ The ability of TD NMR methods to be deployed in situ for porous material synthesis^98,121^ will undoubtedly yield additional applications in the space of hierarchical material synthesis.

A combination of differentiated insights attainable from TD NMR and growing awareness and access to such methodologies heralds a bright future for the field.
